# Uvulopalatopharyngoplasty Versus Expansion Sphincter Pharyngoplasty: A Single Centre Experience

**DOI:** 10.3390/clockssleep7030038

**Published:** 2025-07-29

**Authors:** Teresa Bernadette Steinbichler, Birte Bender, Roland Hartl, Verena Strasser, Daniel Sontheimer, Sladjana Buricic, Barbara Kofler, Birgit Högl, Herbert Riechelmann, Benedikt Hofauer

**Affiliations:** 1 Department of Otorhinolaryngology, Head and Neck Surgery, Medical University of Innsbruck, 6020 Innsbruck, Austria; 2 Department of Neurology, Sleep Disorders Clinic, Medical University of Innsbruck, 6020 Innsbruck, Austria

**Keywords:** polygraphy, obstructive sleep apnoea syndrome, tonsillectomy, adenotomy, tonsillotomy, respiratory disturbance index

## Abstract

Background: Uvulopalatopharyngoplasty (UPPP) and expansion sphincter pharyngoplasty (ESP) are two standard surgical procedures for the treatment of snoring and obstructive sleep apnea. In a retrospective clinical trial, we compared the two surgical techniques regarding objective sleep parameters and patients’ reported outcomes. Materials and Methods: Patients treated with UPPP or ESP between January 2016 and February 2020 were included in this retrospective clinical trial. Pre- and postoperative AHI, BMI, and smoking habits were recorded. Subjective improvement was assessed by the ESS score and symptom relief reported by patients and their bed partners. Results: Between 2016 and 2020, 114 patients were included in the study, 74 patients suffered from OSA, and 30 patients had non-apnoeic snoring (AHI < 5/h). No preoperative sleeping studies were available in 10 patients (10/114; 9%). Based on the findings during drug-induced sedation endoscopy, most patients received an ESP (71/114, 62%), and 43 patients received a UPPP (43/114, 38%). Additionally, in 52/114 (46%), radio frequency ablation of the tongue base was performed if DISE revealed retrolingual collapse. ESP reduced AHI from 21.1 ± 10.8/h to 13.3 ± 12.1/h (*p* = 0.04), whereas UPPP caused a non-significant decrease in the AHI from 25.0 ± 13.8/h to 18.2 ± 14.6/h (*p* = 0.6). A minor secondary bleeding was observed in 32 patients, which was effectively treated with electrocautery or conservative therapy (32/114). This was more common in the ESP group (22/71; 31%) than in the UPPP group (10/43; 23%). Postoperative need for analgesics was higher in the ESP group than in the UPPP group. The ESS score showed no significant improvement after UPPP or ESP (*p* = 0.3), but subjective improvement in snoring was reported by 87/114 (76%) patients. Conclusion: AHI reduction was significantly higher in the ESP patient group than in the UPPP group. ESP patients had a slightly higher rate of minor secondary bleeding and postoperative need for analgesics than UPPP patients.

## 1. Introduction

The rationale of palatal surgery in the management of sleep-disordered breathing is to alleviate retropalatal obstructions during sleep. Flutter of the soft palate is one of the major causes of non-apnoeic snoring. Additionally, the soft palate is commonly involved in upper airway obstruction in obstructive sleep apnea (OSA) as part of a multilevel collapse [1]. During palatal surgery, excessive soft tissue of the palate is removed and placed in a more anterior position [2,3,4]. Uvulopalatopharyngoplasty (UPPP) was traditionally the most widely performed surgical procedure to reduce palatal collapse and vibration [4,5]. It was first described in 1964 by Ikematsu for the treatment of snoring and in 1981 by Fujita and colleagues for the treatment of OSA [6,7]. However, the surgical outcomes of UPPP were inconsistent, and the overall success rate for mild to severe OSA was reported to be between 35 and 50%, according to various studies [8,9]. UPPP is, in general, a painful procedure associated with complications such as nasopharyngeal stenosis and incompetence. Due to these reasons, conservative modifications, such as the modified Fujita technique described by Pirsig and co-authors [10] and non-ablative techniques, have been developed in recent years, leading to a decline in the use of traditional UPPP among sleep surgeons. In recent years, palatal surgery has increasingly focused on barbed techniques that suspend the palatopharyngeus muscle to the pterygomandibular raphe. These have demonstrated a surgical success rate between 50 and 80%, according to recent studies, with a much lower incidence of postoperative comorbidities [8].

Cahali and colleagues reported, in 2004, that treatment of lateral pharyngeal wall collapse is necessary to achieve more positive surgical outcomes [11]. Expansion sphincter pharyngoplasty (ESP) isolates and rotates the palatopharyngeal muscle while leaving the superior pharyngeal constrictor muscle intact, effectively reducing collapse of the lateral pharyngeal wall during sleep [12,13]. The ESP was introduced in 2007 by Pang and Woodson and reported to be more effective than traditional UPPP in selected patients [13].

In our centre, patients are selected for either UPPP or ESP regarding their sleep parameters, especially the apnea-hypopnea index (AHI) and collapse patterns during drug-induced sedation endoscopy (DISE). In this retrospective study, we evaluated the results of UPPP and ESP in terms of objective sleep parameters and subjective daytime sleepiness as measured by the Epworth Sleepiness Scale (ESS) [14]. Furthermore, bed partners were asked for a reduction in symptoms. The current study retrospectively compares ESP and UPPP regarding both objective (AHI, ODI) and subjective (ESS, partner reports) outcomes in a clinical cohort [14]. Given the non-randomized, retrospective design, we also explore potential confounding factors and acknowledge methodological limitations.

## 2. Results

### 2.1. Study Population

Between January 2016 and February 2020, 114 patients received palatal surgery at the Department of Otorhinolaryngology, Medical University of Innsbruck. Most patients were male (98/114, 86%), and the mean (±SD) age was 41.9 (±11.4) years. The mean (±SD) preoperative BMI was 27.8 (±3.6) kg/m^2^. Further patient characteristics are displayed in Table 1.

Patients in the ESP group (47 ± 11 years) were older than those in the UPPP group (36 ± 11 years; *p* < 0.001). In both groups, most patients were male (UPPP: 39/43; 91%; ESP: 59/71; 83%). There was no significant difference in mean BMI in the UPPP and ESP group (*p* = 0.78). Additionally, there was no difference in the distribution of tonsillar size between the two groups (*p* = 0.12; Table 2).

### 2.2. Surgical Procedure

Based on the findings during DISE, most patients received an ESP (71/114, 62%), and 43 patients received a UPPP (43/114, 38%). A tonsillectomy was performed if the patient still had tonsils (104/114; 91%). If the patient had no tonsils at the time of oropharyngeal surgery, a routine ESP was performed. Palatal surgery was combined with a radiofrequency ablation of the tongue base in 46% (52/114) (Table 1). UPPP was combined with radiofrequency ablation of the tongue base in 19/42 (45%) patients, and ESP was combined with radiofrequency ablation of the tongue base in 33/71 (46%) patients. According to Chi’s square test, there was no significant difference in the use of RFT in the UPPP and ESP group (*p* = 0.7).

The most common surgical complication was a dehiscence of the palatal sutures (12%; 14/114). This was more frequent in the UPPP group (16/43; 14%) than in the ESP group (8/71; 11%). A mild secondary bleeding according to a grade A-C described by Sarny and colleagues in 2011 was observed in 32 patients, which was effectively treated with electrocautery or conservative therapy (32/114) [16]. This was more common in the ESP group (22/71; 31%) than in the UPPP group (10/43; 23%). The risk of secondary bleeding was independent of an additional radiofrequency ablation of the tongue base. A secondary bleeding occurred in 17/62 patients without and in 19/52 patients with radiofrequency ablation of the tongue base (*p* = 0.39).

One ESP patient developed pneumonia postoperatively, which was effectively treated with intravenous antibiotics. No other surgical complications, such as nasal regurgitation or stenosis, were observed during follow-up after 12 weeks.

The postoperative need for analgesics was slightly higher in the ESP group than in the UPPP group. In the ESP group, 9/71 (13%) received an analgesic Step III medication, whereas in the UPPP group, 2/43(5%) received an analgesic Step III medication. Step II and Step I pain medications were equally distributed in both groups.

### 2.3. Preoperative Sleeping Parameters

Of the 114 patients, 74 suffered from OSA, and 30 had non-apnoeic snoring (AHI < 5/h) [17]. No preoperative sleeping studies were available in 10 patients (10/114; 9%). Mean preoperative AHI was 15.2 ± 14/h (*n* = 104). Mean preoperative AHI from patients suffering from OSA was 20.2 ± 13.8 (*n* = 74). Mean preoperative ODI was 12.1 ± 12.7/h (*n* = 91). Mean preoperative AHI in the UPPP group (17.1 ± 17.4/h; *n* = 36) and mean preoperative AHI in the ESP group did not differ significantly (15.9 ± 11.1; *n* = 68; *p* = 0.7).

### 2.4. Postoperative Sleeping Parameters

**Figure 1:**
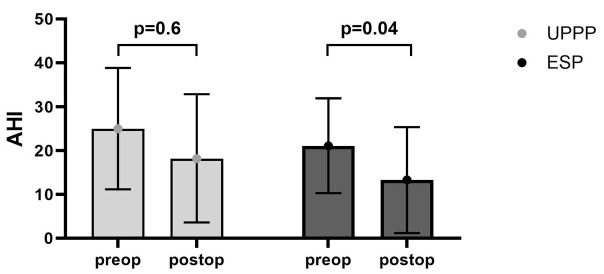
Pre- and postoperative AHI after either UPPP or ESP. Mean, overall preoperative AHI was reduced from 20.2 ± 13.8/h to 16.8 ± 19.1/h (*n* = 74; *p* = 0.07). ESP caused a decrease in the preoperative AHI from 21.1 ± 10.8/h to 13.3 ± 12.1/h (*p* = 0.04). UPPP caused a non-significant decrease in the preoperative AHI from 25.0 ± 13.8/h to 18.2 ± 14.6/h (*p* = 0.6).

Postoperative polygraphy or polysomnography was only performed in patients suffering from OSA (*n* = 74), approximately 12 weeks after surgery. Mean preoperative AHI was reduced from 20.2 ± 13.8/h to 16.8 ± 19.1/h (*n* = 74; *p* = 0.07). ESP caused a decrease in the preoperative AHI in OSA patients, from 21.1 ± 10.8/h to 13.3 ± 12.1/h (*p* = 0.04). UPPP caused a non-significant decrease in the preoperative AHI in OSA patients from 25.0 ± 13.8/h to 18.2 ± 14.6/h (*p* = 0.6; Figure 1). To further test for differences in the success of the two surgery types, the ΔAHI (pre- to post-AHI difference) was calculated. Mean ΔAHI was 14.6 ± 6.4/h in the ESP group and 11.6 ± 7.7/h in the UPPP group (*p* = 0.03).

However, a combination of palatal surgery with radiofrequency ablation of the tongue base was effective in reducing preoperative AHI in OSA patients (23.5 ± 12.8/h to 14.9 ± 12.9/h; *n* = 22; *p* = 0.03). Subgroup analysis revealed no statistically significant difference in mean pre- and postoperative AHI in the UPPP/ESP group with or without radiofrequency ablation of the tongue base (Table 1).

Patients with moderate or severe OSA particularly profited from palatal surgery (r = 0.4; *p* = 0.007).

Of the patients who had no tonsils at the time of palatal surgery, two had non-apneic snoring (2/10; 20%), 7 suffered from OSA (7/10; 70%), and for one patient (1/10; 10%), no preoperative sleeping study was available. Mean preoperative AHI from patients with OSA and tonsillectomy was 20.4 ± 13.5/h (*n* = 67), and mean preoperative AHI from patients with OSA and without tonsillectomy was 33.5 ± 16.5/h (*n* = 7). The postoperative AHI was 17.2 ± 16.9/h in the tonsillectomy group, compared to 10.0 ± 6.0/h in the group without tonsillectomy, indicating that palatal surgery might also be effective in patients who have previously had their tonsils removed.

Smoking habits did not influence the postoperative AHI (*p* = 0.23), but a BMI > 25 kg/m^2^ had a negative influence on postoperative AHI (*p* = 0.003).

Mean postoperative ODI was 10.2 ± 13.7/h (44/114) in all patients. According to the literature on OSA treatment, Sher’s criteria of a 50 % reduction in AHI and/or an AHI less than 20 are reported to be the benchmark for successful surgery [18]. These criteria were successfully fulfilled in 38% of the patients (ESP: 41%; UPPP: 27%; Table 3).

### 2.5. Patient Reported Outcome

Subjective improvement of OSA-associated symptoms was evaluated by the ESS score [14]. The mean preoperative ESS Score was available in 89 patients and was 9.0 ± 5.0 [14]. The mean preoperative ESS Score in OSA patients was 9.8 ± 5.5 (*n* = 35). The mean postoperative ESS Score was 7.8 ± 5.2, but it was only available in 27 of the OSA patients (*p* = 0.15), which substantially limits the significance of this result.

Patients and their bed partners were further asked about improvement in snoring habits. Subjective improvement in snoring was reported by 87/114 (76%) patients. A survey of bed partners was available in 35 patients. Subjective improvement in snoring was reported in 26/35 (74%) patients, and 7 of their bed partners returned to a common bedroom after surgery.

## 3. Discussion

The prevalence of OSA in the general population is between 9 and 38% and is higher in men [15]. Snoring is one of the most obvious clinical signs of OSA, but can be an impairment by itself, especially for the bed partners. The incidence of snoring increases with age and BMI and is reported to be 45% in male patients and 30% in female patients older than 65 [19]. The standard treatment for OSA is non-invasive continuous positive airway pressure (CPAP) therapy. Although CPAP is very effective in reducing AHI, it is not well tolerated by all patients. When adherence is defined as the use of CPAP for more than 4 h per night for more than 70% of nights, CPAP adherence rates of 75% have been reported. A much smaller percentage of patients use CPAP throughout their entire sleep [20,21]. A good alternative to CPAP in non-obese patients is oropharyngeal surgery if the AHI is below 30/h (mild/moderate OSA) and the obstruction is located at the level of the oropharynx/tonsils. Validated surgical treatments for snoring and OSA include UPPP and ESP [9].

They are effective in treating retropalatal and oropharyngeal obstructions and reduction of palatal flutter, which is the main cause for non-apneic snoring [2,3,4,5,22]. However, the majority of OSA patients (91%) have multilevel collapse, limiting the success of selected surgical procedures [23]. In this context, nearly one fourth of the patients also have collapse of the lateral pharyngeal wall, which is insufficiently treated by UPPP [23,24]. ESP also addresses this collapse of the lateral pharyngeal wall by rotating the palatopharyngeus muscle [13] and might therefore be superior to traditional UPPP [12,13]. According to the results published by Pang et al. in 2007, ESP was more effective in reducing preoperative AHI than UPPP [12,13]. In our study group, ESP reduced AHI from 21.1 ± 10.8/h to 13.3 ± 12.1/h (*p* = 0.04), whereas UPPP caused a non-significant decrease in the preoperative AHI from 25.0 ± 13.8/h to 18.2 ± 14.6/h (*p* = 0.6), supporting the results from Pang and colleagues. These data might be biased by the fact that we tend to prefer ESP over UPPP if DISE revealed complete concentric collapse of the velum or lateral pharyngeal wall collapse.

The retrospective design and absence of randomization likely introduce selection bias, as surgical technique was chosen based on individual DISE findings and clinical judgment.

The ESP group was significantly older than the UPPP group. Age-related differences may have influenced pain perception, healing responses, and possibly treatment effect.

In some patients, different diagnostic tools were used pre- and postoperatively (polysomnography, polygraphy, and WatchPat), leading to heterogeneity in AHI scoring.

Postoperative sleeping studies were performed in nearly 50% of the patients with WatchPat analysis (*n* = 35), where preoperative sleeping studies were only performed in 14% (*n* = 15), with WatchPat analysis adding significance to these results, as WatchPat in particular is known to potentially overestimate AHI in mild OSA [25].

In this study cohort, some patients (*n* = 14) with severe OSA were treated with oropharyngeal surgery, which is not in line with current guidelines [9]. These patients were selected for UPPP/ESP if they did not tolerate CPAP at all, had an exceptionally strong desire for surgery, and—provided the anatomical conditions were suitable—after a thorough explanation that surgery alone would probably not be sufficient. In addition, some of these patients had undergone tonsillectomy for another reason (recurrent tonsillitis) and asked for additional oropharyngeal surgery due to known OSA.

Whereas the study by Pang and colleagues found the complication rate to be equal in UPPP and ESP, we observed a slightly higher rate of mild secondary bleeding in ESP patients. We explain this by the more lateral dissection in the tonsillar pillar during ESP due to the isolation of the palatopharyngeal muscles and the associated exposure of the vessels underlying these muscles, whose caliber size tends to increase laterally [14,21]. This also explains the higher rate of postoperative bleeding compared to conventional tonsillectomy [14]. Additionally, ESP patients also had a higher need for postoperative analgesics in our study cohort, as postoperative pain is due to damage to the tonsillar fossa musculature, which is routinely left intact during traditional UPPP [26]. No long-term complications, such as velopharyngeal insufficiency or velar stenosis, were noted, which were historically associated with ESP and UPPP [13]. It is noteworthy that in recent years, new palatal surgical techniques for snoring and OSAS have been developed that also address the lateral pharyngeal wall collapse and laterally expand the oropharyngeal inlet, e.g., barbed reposition pharyngoplasty or anterolateral advancement pharyngoplasty [27,28]. Both techniques were described to be equally efficient as ESP in decreasing preoperative AHI but might be associated with less pain, less dysphagia, and less foreign body sensation at the throat postoperatively. Compared to ESP, they might be faster and simpler techniques, with minimal blood loss and better preservation of mucosal and muscle tissue [29,30].

In the context of multilevel collapse in OSA patients, more than 80% of these patients suffer from additional tongue base collapse during apneas/hypopneas [26]. Consistent with these results, radiofrequency ablation of the tongue base also caused a significant additional decrease in the AHI (23.5 ± 12.8/h to 14.9 ± 12.9/h; *n* = 22; *p* = 0.03) in our study cohort, confirming the need for multilevel surgery in OSA patients.

Sher’s criteria of 50 % reduction in AHI and/or an AHI less than 20 are reported to be the benchmark for successful surgery [18]. These criteria were successfully fulfilled in 38% of the patients (ESP: 41%; UPPP: 27%; Table 2). Sher’s criteria are widely used and reflect everyday clinical practice. However, they are not very high and are based solely on numerical PSG values. These criteria are often criticized as inadequate to adequately capture the clinical benefit, as these thresholds are based solely on numerical AHI changes and do not reflect quality of life or subjective improvements, such as snoring reduction [8].

The ESS score, as a tool for measuring daytime sleepiness, showed no significant improvement after UPPP or ESP (*p* = 0.3). The postoperative ESS score was only available in 27 patients, limiting the results [14]. However, most patients (76%) and their bed partners (74%) reported relief from snoring and a subjective improvement in sleep, and seven bed partners returned to a common bedroom.

## 4. Materials and Methods

All patients treated with UPPP or ESP at the Department of Otorhinolaryngology, Head and Neck Surgery, Medical University of Innsbruck between January 2016 and February 2020, were consecutively included in this retrospective clinical trial. A positive vote of the Ethical Committee of the Medical University of Innsbruck was obtained (AN 2020–1290/201). Due to the retrospective character of the study, a written informed consent from the patient was not necessary.

All patients underwent a standard basic clinical examination, which included evaluation of BMI, daytime sleepiness using the Epworth Sleepiness Scale [14], and endoscopy of the nasal cavity, nasopharynx, oral cavity, oropharynx, hypopharynx, and larynx. Respiratory events during sleep were recorded preoperatively with cardiorespiratory polygraphy (*n* = 36), Watchpat (*n* = 15), or polysomnography (*n* = 53). In non-apnoeic snorers, OSA was excluded with cardiorespiratory polygraphy (*n* = 15), Watchpat (*n* = 7), or polysomnography (*n* = 8). Postoperative respiratory events were recorded with cardiorespiratory polygraphy (*n* = 15), Watchpat (*n* = 35), or polysomnography (*n* = 24). Of note, the use of these different diagnostic tools introduces potential heterogeneity in sleep parameter measurements.

If available, bed partners were questioned for symptoms such as snoring and apneas.

All patients received a DISE either alone or as the first step of the palatal surgery under propofol sedation for the evaluation of pharyngeal collapse patterns using a flexible fiberoptic nasopharyngoscope. The level (palate, oropharynx, tongue base, and epiglottis), the direction (anteroposterior, concentric, and lateral), and the degree of collapse (none, partial, or complete) were scored in standard fashion with the VOTE score [31]. Patient allocation to surgical procedures was based on individual collapse patterns identified during drug-induced sedation endoscopy (DISE), without randomization.

After surgery, patients were hospitalized for 5 days for pain management and inpatient surveillance. They were controlled every day for the first 5 days for wound dehiscence, infections, or signs of bleeding. All patients were routinely controlled after 3 months postoperatively in our outpatient department.

### 4.1. Surgical Technique

**Figure 2:**
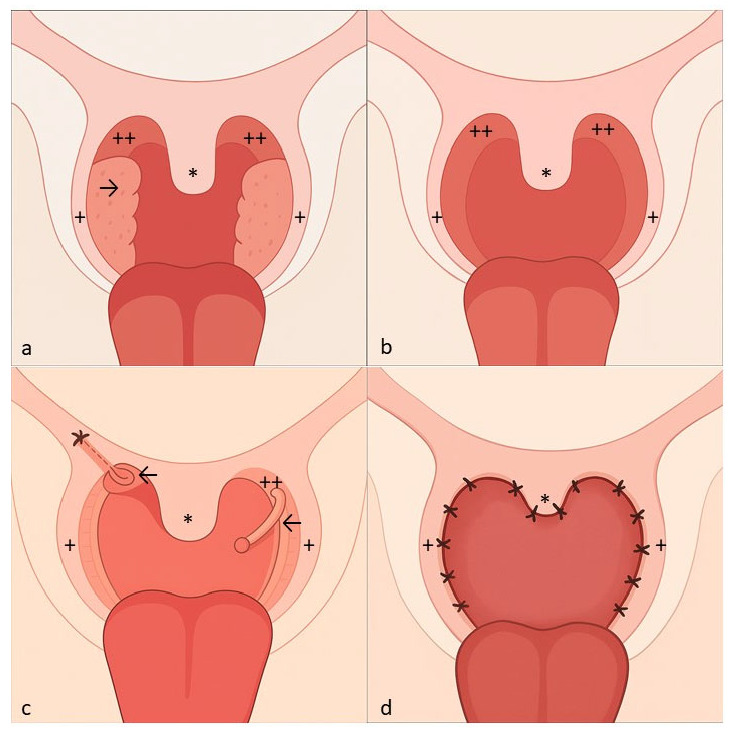
Surgical Procedure. (**a**) Situs before surgery. (**b**) Both ESP and UPPP started with a bilateral tonsillectomy by cold dissection when tonsils (→) were still present. (**c**) In ESP, the palatopharyngeal muscles (←) on both sides were dissected. After horizontal transection of this muscle inferiorly, a submucous tunnel was made from the apex of the tonsillar fossa by blunt dissection. The palatopharyngeal muscles were rotated through this tunnel superolaterally with the fixation of their inferior muscle bulk near the hamulus pterygoideus by Vicryl 2-0. This step was skipped in UPPP and only redundant velar tissues from the free margin of the soft palate, tonsillar pillars, and uvula (*) were excised without resecting the muscles of the velum. (**d**) As the final step of both surgical techniques, the tonsillar pillars were apposed with Vicryl 2-0. (Annotations: * Uvula, → Tonsils, ← M. palatopharyngeus, + anterior tonsillar pillar, ++ posterior tonsillar pillar).

**Figure 3:**
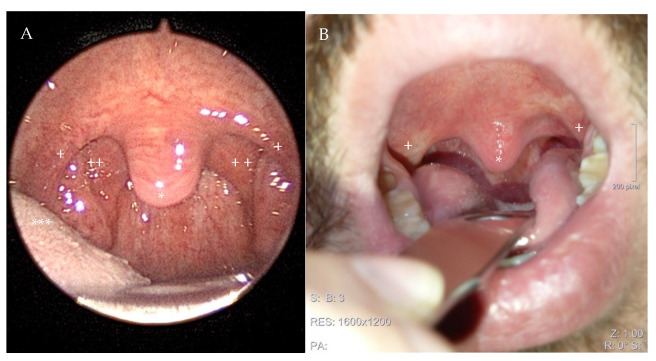
Image of a 38-year-old male patient before (**A**) and twelve weeks after expansion sphincter pharyngoplasty (ESP). Thepostoperative picture demonstrates the increased velar distance from the posteior pharyngeal wall due to the removal of excess velar tissue and an enlargement of the lateral oropharyngeal space due to the rotation of the palatopharyngeal muscle (**B**). The preoperative AHI of 28/h decreased to 12/h at outpatient polygraphy 12 weeks after ESP, and the patient described a decrease in snoring and his daytime symptoms. (Annotaions: + anterior tonsillar pillar, ++ posterior tonsillar pillar, * uvula, *** tongue).

All surgical procedures were performed by two surgeons (T.B.S.; B.B.) under general anaesthesia with the patient in supine position and the head in hyperextension. A McIvor mouth gag was used to expose the oropharynx. The operation started with a bilateral tonsillectomy by cold dissection when the tonsils were still present. We used the modification of the standard UPPP suggested by Fujita in 1984 [6]. Redundant velar tissues from the free margin of the soft palate, tonsillar pillars, and uvula were excised without resecting the muscles of the velum (Figure 2 and Figure 3A,B).

In ESP, the palatopharyngeal muscles on both sides were dissected. After horizontal transection of this muscle inferiorly, a submucous tunnel was made from the apex of the tonsillar fossa by blunt dissection. The palatopharyngeal muscles were rotated through this tunnel superolaterally with fixation of their inferior muscle bulk near the hamulus pterygoideus by Vicryl 2–0 (Figure 2) [12]. As the final step of both surgical techniques, the tonsillar pillars were apposed with Vicryl 2–0. If necessary, the uvula length was reduced (Figure 2).

Radiofrequency ablation of the tongue base was performed if DISE revealed retrolingual collapse or if tongue base hyperplasia was noted. For the radiofrequency ablation of the tongue base, we used the Celon device (Olympus, Hamburg, Germany) with 8 watts and applied 8–10 lesions [32].

All patients received single-shot antibiotics with amoxicillin/clavulanate 2.2 g intravenously 30 min before surgery. Postoperative pain management on days 1–5 was standardized in three steps. All patients received paracetamol 4  ×  1 g  +  naproxen 2  ×  500 mg as step I medication. If the patient rated pain on a pain scale (0  =  no pain; 10   =   maximum conceivable pain) higher than 3, the treatment level was increased one step. Step II medication consisted of step I, plus additional modified-release hydromorphone 2 × 2 mg. For step III pain medication, 2 × 4 mg hydromorphone was added. Correspondingly, if the pain was scored below 4, pain medication was decreased one step [33].

### 4.2. Data Analysis

Interval data were presented as mean ± standard deviation (SD) unless indicated otherwise and tested with the paired samples t-test. Categorical data were analyzed with the McNemar test. Statistical analysis was performed using SPSS 22 (IBM Corporation, Armonk, NY, USA). No formal power calculation was conducted, and subgroup comparisons may be underpowered.

## 5. Conclusions

AHI reduction was significantly higher in the ESP patient group than in the UPPP group, especially if it was combined with radiofrequency ablation of the tongue base. ESP patients had a slightly higher rate of minor secondary bleedings and postoperative need for analgesics than UPPP patients. However, the study did not include a priori or post-hoc power calculations. Some subgroup analyses (e.g., the effect of RFT or prior tonsillectomy) may have been underpowered, limiting the strength of the conclusions.

## Figures and Tables

**Table 1:** The characteristics of 114 patients treated with palatal surgery for obstructive sleep apnea and/or snoring at the Department of Otorhinolaryngology, Medical University of Innsbruck, between 2016 and 2020. Abbreviations: AHI: Apnea-Hypopnea Index per hour, BMI: body mass index, ESP: Expansion Sphincter Pharyngoplasty, UPPP: Uvulopalatopharyngoplasty.

Variable	Value	Count	Percent
Sex	female	16	14%
	male	98	86%
Age	≤30	21	18%
	31–40	30	26%
	41–50	38	33%
	≥51	25	22%
BMI (in kg/m^2^)	underweight (<18.5 kg/m^2^)	2	2%
	normal (18.5–25 kg/m^2^)	18	16%
	Overweight (25–30 kg/m^2^)	64	56%
	Obese (>30 kg/m^2^)	30	26%
Smoking	yes	46	40%
	no	60	53%
	missing	8	7%
OSA-severity	Non (AHI * < 5/h)	30	26%
	Mild (AHI 5–15/h)	34	30%
	Moderate (AHI 15–30/h)	26	23%
	Severe (AHI > 30/h)	14	12%
	Missing	10	9%
Surgical procedure	UPPP	43	38%
	ESP	71	62%
Radiofrequency ablation of the tongue base	yes	52	46%
	no	62	54%
tonsillectomy	yes	104	91%
	no	10	9%

* Apnoe-Hypopnoe-Index per hour as classified by the American Academy of Sleep Medicine [15].

**Table 2:** Tonsillar size and BMI in the UPPP and ESP group. Abbreviations: BMI: body mass index, ESP: Expansion Sphincter Pharyngoplasty, UPPP: Uvulopalatopharyngoplasty.

Variable	Value	UPPP (*n* = 43)	ESP (*n* = 71) *
Tonsillar size (Brodksy classification)	0	1 (2%)	2 (3%)
	1	14 (33%)	24 (34%)
	2	21 (49%)	28 (39%)
	3	5 (12%)	4 (6%)
	4	2 (5%)	3 (4%)
BMI (in kg/m^2^)	underweight (<18.5 kg/m^2^)	1 (2%)	1 (1%)
	normal (18.5–25 kg/m^2^)	9 (21%)	9 (13%)
	Overweight (25–30 kg/m^2^)	21 (49%)	43 (60%)
	Obese (>30 kg/m^2^)	12 (28%)	18 (25%)

* in the ESP group, 10 patients had no tonsils at the time of surgery.

**Table 3:** Comparison of pre- and postoperative sleep parameters in patients treated with either UPPP or ESP. Only ESP caused a significant decrease in preoperative AHI. Surgical success was further evaluated by the fulfillment of Sher’s criteria and was achieved in 41% of patients treated with ESP and in 27% treated with UPPP. Abbreviations: AHI: Apnea-Hypopnea Index per hour; ESP: Expansion Sphincter Pharyngoplasty; UPPP: Uvulopalatopharyngoplasty.

	ESP	UPPP
total patient count	71	43
AHI * preoperative	21.1 ± 10.8/h	25.0 ± 13.8/h
AHI * postoperative	13.3 ± 12.1/h	18.2 ± 14.6/h
*p* value	0.04	0.6
success rate **	41%	27%

* Apnoe-Hypopnoe-Index per hour as classified by the American Academy of Sleep Medicine [15]. ** Success rate according to Sher’s criteria of a 50 % reduction in AHI and/or an AHI less than 20, which was reported to be the benchmark for successful surgery [18].

## Data Availability

The data will be made available upon reasonable request. My manuscript has no associated data.

## References

[1] Vlad A.M., Stefanescu C.D., Stefan I., Zainea V., Hainarosie R. (2023). Comparative Efficacy of Velopharyngeal Surgery Techniques for Obstructive Sleep Apnea: A Systematic Review. Medicina.

[2] Powell N.B., Riley R.W., Robinson A. (1998). Surgical management of obstructive sleep apnea syndrome. Clin. Chest Med..

[3] Riley R.W., Powell N.B., Guilleminault C., Clerk A., Troell R. (1995). Obstructive sleep apnea. Trends in therapy. West. J. Med..

[4] Steinbichler T.B., Bender B., Giotakis A.I., Dejaco D., Url C., Riechelmann H. (2018). Comparison of two surgical suture techniques in uvulopalatopharyngoplasty and expansion sphincter pharyngoplasty. Eur. Arch. Otorhinolaryngol..

[5] Friedman M. (2009). Sleep Apnea and Snoring: Surgical and Non-Surgical Therapy.

[6] Fujita S., Conway W., Zorick F., Roth T. (1981). Surgical correction of anatomic azbnormalities in obstructive sleep apnea syndrome: Uvulopalatopharyngoplasty. Otolaryngol. Head Neck Surg..

[7] Ikematsu T. (1964). Study of snoring. Therapy. J. Jpn. Otol. Rhinol. Laryngol. Soc..

[8] Iannella G., Pace A., Magliulo G., Vicini C., Lugo R., Vanderveken O.M., de Vries N., Pang K., Thuler E., Jacobowitz O. (2024). International expert consensus statement: Surgical failure in obstructive sleep apnea. Sleep. Breath..

[9] Stuck B.A., Arzt M., Fietze I., Galetke W., Hein H., Heiser C., Herkenrath S.D., Hofauer B., Maurer J.T., Mayer G. (2020). Teil-Aktualisierung S3-Leitlinie Schlafbezogene Atmungsstörungen bei Erwachsenen. Somnologie.

[10] Pirsig W., Schafer J., Yildiz F., Nagel J. (1989). Uvulopalatopharyngoplasty without complications: A Fujita complication. Laryngorhinootologie.

[11] Cahali M.B. (2003). Lateral pharyngoplasty: A new treatment for obstructive sleep apnea hypopnea syndrome. Laryngoscope.

[12] Pang K.P., Pang E.B., Win M.T., Pang K.A., Woodson B.T. (2016). Expansion sphincter pharyngoplasty for the treatment of OSA: A systemic review and meta-analysis. Eur. Arch. Otorhinolaryngol..

[13] Pang K.P., Woodson B.T. (2007). Expansion sphincter pharyngoplasty: A new technique for the treatment of obstructive sleep apnea. Otolaryngol. Head Neck Surg..

[14] Johns M.W. (1991). A new method for measuring daytime sleepiness: The Epworth sleepiness scale. Sleep.

[15] Senaratna C.V., Perret J.L., Lodge C.J., Lowe A.J., Campbell B.E., Matheson M.C., Hamilton G.S., Dharmage S.C. (2016). Prevalence of obstructive sleep apnea in the general population: A systematic review. Sleep. Med. Rev..

[16] Sarny S., Ossimitz G., Habermann W., Stammberger H. (2011). Hemorrhage following tonsil surgery: A multicenter prospective study. Laryngoscope.

[17] Ruehland W.R., Rochford P.D., O’Donoghue F.J., Pierce R.J., Singh P., Thornton A.T. (2009). The new AASM criteria for scoring hypopneas: Impact on the apnea hypopnea index. Sleep.

[18] Pang K.P., Rotenberg B.W. (2016). The SLEEP GOAL as a success criteria in obstructive sleep apnea therapy. Eur. Arch. Otorhinolaryngol..

[19] Mendes F.A., Marone S.A., Duarte B.B., Arenas A.C. (2014). Epidemiologic profile of patients with snoring and obstructive sleep apnea in a university hospital. Int. Arch. Otorhinolaryngol..

[20] Pepin J.L., Woehrle H., Liu D., Shao S., Armitstead J.P., Cistulli P.A., Benjafield A.V., Malhotra A. (2018). Adherence to Positive Airway Therapy After Switching From CPAP to ASV: A Big Data Analysis. J. Clin. Sleep. Med..

[21] Veasey S.C., Rosen I.M. (2019). Obstructive Sleep Apnea in Adults. N. Engl. J. Med..

[22] Sommer U.J., Heiser C., Gahleitner C., Herr R.M., Hormann K., Maurer J.T., Stuck B.A. (2016). Tonsillectomy with Uvulopalatopharyngoplasty in Obstructive Sleep Apnea. Dtsch. Arztebl. Int..

[23] Vanderveken O.M., Maurer J.T., Hohenhorst W., Hamans E., Lin H.S., Vroegop A.V., Anders C., de Vries N., Van de Heyning P.H. (2013). Evaluation of drug-induced sleep endoscopy as a patient selection tool for implanted upper airway stimulation for obstructive sleep apnea. J. Clin. Sleep. Med..

[24] Remmers J.E., deGroot W.J., Sauerland E.K., Anch A.M. (1978). Pathogenesis of upper airway occlusion during sleep. J. Appl. Physiol. Respir. Environ. Exerc. Physiol..

[25] Gan Y.J., Lim L., Chong Y.K. (2017). Validation study of WatchPat 200 for diagnosis of OSA in an Asian cohort. Eur. Arch. Otorhinolaryngol..

[26] Kim J.S., Kwon S.H., Lee E.J., Yoon Y.J. (2017). Can Intracapsular Tonsillectomy Be an Alternative to Classical Tonsillectomy? A Meta-analysis. Otolaryngol. Head Neck Surg..

[27] Maniaci A., Di Luca M., Lechien J.R., Iannella G., Grillo C., Grillo C.M., Merlino F., Calvo-Henriquez C., De Vito A., Magliulo G. (2022). Lateral pharyngoplasty vs. traditional uvulopalatopharyngoplasty for patients with OSA: Systematic review and meta-analysis. Sleep. Breath..

[28] Vicini C., Hendawy E., Campanini A., Eesa M., Bahgat A., AlGhamdi S., Meccariello G., DeVito A., Montevecchi F., Mantovani M. (2015). Barbed reposition pharyngoplasty (BRP) for OSAHS: A feasibility, safety, efficacy and teachability pilot study. “We are on the giant’s shoulders”. Eur. Arch. Otorhinolaryngol..

[29] Emara T.A., Elmonem M., Khaled A.M., Genedy H.A.H., Youssef R.S. (2024). Anterolateral advancement pharyngoplasty versus barbed reposition pharyngoplasty in patients with obstructive sleep apnea. Eur. Arch. Otorhinolaryngol..

[30] Emara T.A., Hassan M.H., Mohamad A.S., Anany A.M., Ebrahem A.E. (2016). Anterolateral Advancement Pharyngoplasty: A New Technique for Treatment of Obstructive Sleep Apnea. Otolaryngol. Head Neck Surg..

[31] De Vito A., Carrasco Llatas M., Ravesloot M.J., Kotecha B., De Vries N., Hamans E., Maurer J., Bosi M., Blumen M., Heiser C. (2018). European position paper on drug-induced sleep endoscopy: 2017 Update. Clin. Otolaryngol..

[32] Stuck B.A., Kopke J., Hormann K., Verse T., Eckert A., Bran G., Duber C., Maurer J.T. (2005). Volumetric tissue reduction in radiofrequency surgery of the tongue base. Otolaryngol. Head Neck Surg..

[33] Bender B., Blassnigg E.C., Bechthold J., Kral F., Riccabona U., Steinbichler T., Riechelmann H. (2015). Microdebrider-assisted intracapsular tonsillectomy in adults with chronic or recurrent tonsillitis. Laryngoscope.

